# Evaluation of a new model of care for bladder management in a statewide spinal cord service

**DOI:** 10.1038/s41393-024-01059-5

**Published:** 2025-01-15

**Authors:** Belinda J. Gabbe, Stacey RJ Haughton, Andrew Nunn, Marnie Graco, Chris Michael, Sandra Reeder, Rebekah McGaw, David J. Berlowitz

**Affiliations:** 1 School of Public Health and Preventive Medicine, Monash University, Melbourne, VIC Australia; 2 Population Data Science, Swansea University, Swansea, UK; 3 Physiotherapy Department, Austin Health, Melbourne, VIC Australia; 4 Victorian Spinal Cord Service, Austin Health, Heidelberg, VIC Australia; 5 Prosthetics & Orthotics Service, Alfred Health, Melbourne, VIC Australia; 6 Information Technology, Monash University, Melbourne, VIC Australia; 7 Department of Electrical and Computer Systems Engineering, Monash University, Melbourne, VIC Australia; 8 Institute for Breathing and Sleep, Austin Health, Heidelberg, VIC Australia; 9 Department of Physiotherapy, School of Health Sciences, University of Melbourne, Melbourne, VIC Australia; 10 Monash Centre for Health Research and Implementation, Monash University, Melbourne, VIC Australia; 11 Present Address: Department of Physiotherapy, School of Health Sciences, University of Melbourne, Melbourne, VIC Australia

**Keywords:** Rehabilitation, Epidemiology

## Abstract

**Study design:**

Registry-based cohort study.

**Objectives:**

To evaluate the impact of the introduction of a new bladder management model of care at the Victorian Spinal Cord Service (VSCS) on the incidence of subsequent emergency department presentations and readmissions to hospital for urinary tract infection (UTI) in the first 2 years after injury.

**Setting:**

VSCS, Austin Health, Melbourne, Australia.

**Methods:**

A new model of care that prioritized intermittent self-catheterization was implemented at the VSCS on 1 August 2017. Data from the Victorian State Trauma Registry and Austin Health medical record were used to compare the rate of readmissions, emergency department (ED) presentations and hospitalisations for UTI in the first two years post-injury before and after practice was changed.

**Results:**

A total of 333 cases were included; 149 cases pre-model of care change and 184 cases after. 143 males and 41 females with a mean (SD) age of 48.9 (19.7) were admitted to the VSCS following the change in model of care. The rate of any subsequent hospitalisation for UTI (ED presentation or admission) was lower following the introduction of the new bladder management model of care (Incidence rate ratio 0.30, 95% CI 0.12–0.73).

**Conclusions:**

Our data demonstrates the real-world impact of a change in bladder management after new SCI. These data strengthen the consensus recommendation in current practice guidelines.

## Introduction

Bladder and bowel care is often a time-consuming long-term challenge for a person living with a spinal cord injury (SCI) regardless of injury level [1]. People living with SCI frequently require bladder management strategies because of a neurogenic bladder. However, bladder management can cause complications such as urinary tract infections (UTI), a recurrent problem for up to 60% of people living with a SCI [2, 3] and 40% of these individuals report that UTIs have a moderate to severe impact on their life [2]. Patients are at risk of developing septicaemia due to a UTI and in the first year after injury the primary cause for emergency department (ED) admissions is septicemia, disorders of the urethra and urinary tract (primarily UTIs) [4, 5]. Urinary tract infections are the second most common cause of death in people living with SCI [6]. Better bladder and bowel function rates highly amongst health priorities for people living with SCI, while urinary incontinence and urinary tract infection (UTI) are among the most distressing complaints that impact quality of life (QoL) [7, 8]. A previous population-based study of 548 incident SCI cases admitted to Victorian hospitals found that 35% of people were readmitted to hospital for a SCI related condition in the first 2 years after injury [9]. Of the people readmitted, 26% involved a urological complication. The most common readmission reason was a UTI, with the total cost of UTI readmissions and ED visits exceeding $3.3 million Australian dollars over the two year period [9].

There are many methods for managing a neurogenic bladder. Two common options are the use of an indwelling catheter (IDC) and intermittent self-catherisation (ISC). An IDC is inserted into the bladder and can remain in situ for weeks at a time. ISC involves emptying the bladder at a specified, intermittent frequency by person inserting a catheter, completely draining the bladder and then removing the catheter [10, 11]. Whilst the evidence is low, some studies have found that ISC can reduce chronic urogenital complications such as UTI [12–14], and the European Association of Urology (EAU) and American Urological Association (AUA) guidelines recommend the use of ISC over IDC for bladder management [11, 15]. Intermittent self-catheterisation is therefore recommended as the gold standard of care for bladder management [11, 16–18]. The use of IDCs previously had been standard bladder care at the Victorian Spinal Cord Service (VSCS). A retrospective review of bladder management practice at the VSCS found that ISC was associated with a lower rate of UTI in people with SCI [16]. As such, in August 2017 the VSCS introduced a new method of bladder management, ISC. Therefore, the change in practice is important to reduce the risk and rate of UTI and improve QoL for people living with SCI. The aim of this project was to evaluate the impact of a new bladder management strategy following a change from using IDC as standard care to ISC.

## Methods

### Setting

The VSCS is the primary care centre for people with spinal cord injuries in Victoria, Australia.

### Study design and participants

A registry-based cohort study of people with SCI managed before or after the introduction of the new model of bladder management care at the VSCS was conducted. Adult (>15 years) cases of traumatic SCI with a date of injury from 1 July 2014 to 30 June 2020, and acute admission managed at the VSCS, were included. The research was conducted in compliance with the National Health and Medical Research Council of Australia’s 2023 National Statement on Ethical Conduct in Human Research. Ethics approval for this study was granted by the Austin Health Human Research Ethics Committee (Project ID 78300) and the Monash University Human Research Ethics Committee (Project ID 29749). The research was conducted with approval of a waiver of consent as existing data were used.

### Procedures

Eligible cases were identified using the VSTR, the State’s population-based registry for major trauma. Data extracted from the registry included demographic information, cause (transport, fall or other) and intent of injury (intentional, unintentional, intent cannot be determined), pre-existing conditions (Charlson Comorbidity Index), nature of the SCI, the Injury Severity Score (ISS), management in the State’s trauma system (managed at the VSCS or other trauma service, inter-hospital transfer status, intensive care unit admission status), in-hospital outcomes source of funding for the admission. Data related to readmission, hospitalization and ED presentation were extracted from the Austin Health electronic medical record (EMR).

The Charlson Comorbidity Index (CCI) is used to measure mortality risk and burden of disease [19, 20]. It can be used to prognosticate patient long-term mortality [20]. The quintile of socioeconomic status was measured by applying the Index of Relative Socioeconomic Advantage and Disadvantage (IRSAD) to the patient’s postcode of residence. Range included 1 (most disadvantaged) to 5 (most advantaged) [21]. Remoteness of residence was assessed by applying the Accessibility/Remoteness Index of Australia (ARIA) which classifies the patient’s postcode of residence into 1 of 5 categories including major city, inner regional, outer regional, remove and very remote [22]. These categories were consolidated into 2 categories; major cities, and inner regional, outer regional and remote. UTI was defined using the ICD-10-AM diagnosis code, N39.0, for the admission or ED presentation.

Management strategies of bladder care included IDCs that were changed every 6 weeks. They were changed earlier if they became blocked or the patient was diagnosed with a UTI. No antibiotics were administered prior to routine changes [15, 23].

Intermittent catheterisation was completed by either the nursing staff or patient while in hospital. If completed by nursing staff a sterile technique was performed however if a patient completed the IC a clean technique was used. No catheters were re-used. Patients had the option to utilise a self-contained unit [11].

The outcomes of interest for this study included:

i. Readmission (yes/no) and number of readmissions to hospital for UTI in the first 2 years post-injury.
ii. ED presentation (yes/no) and number of subsequent presentations to ED for UTI in the first 2 years post-injury.
iii. Hospitalisation (yes/no) and number of hospitalisations (ED or readmission) for UTI in the first 2 years post-injury.

The outcome of “hospitalisation” included both subsequent ED presentations and hospital readmissions at the Austin. For all analyses, ED presentations excluded the cases of hospital readmission that were via an ED; ED presentations were only where the person was managed and directly discharged from the Austin ED.

### Statistical analysis

For analysis, change in the ‘pre’ model of care commenced 1 August 2017 and ended on 30 April 2017 to remove any potential confounding from early cases, where the new model of care was piloted, between 1 May and 30 July 2017. Frequencies and percentages were used to summarise categorical variables. Median values and interquartile range (IQR) or means with standard deviation (SD) were used to describe continuous data. Baseline variables were compared between model of care groups using t tests if continuous data were normally distributed, Mann–Whitney u tests for non-normally distributed continuous variables, and chi-square tests for categorical variables.

The number of readmissions to hospital, and presentations to ED, for UTI in the first 2 years post-injury were modelled using a negative binomial model to allow for the overdispersion in the data. The model was adjusted for differences in the case-mix on acute admission and an indicator variable created to account for the small proportion of cases where a full 2 years of follow-up had not yet been reached (*n* = 16). Adjusted incidence ratios (IRR) along with the corresponding 95% confidence intervals (CI) were reported. All analyses were performed using Stata Version 17. A *p*-value < 0.05 was considered significant.

## Results

**Fig. 1:**
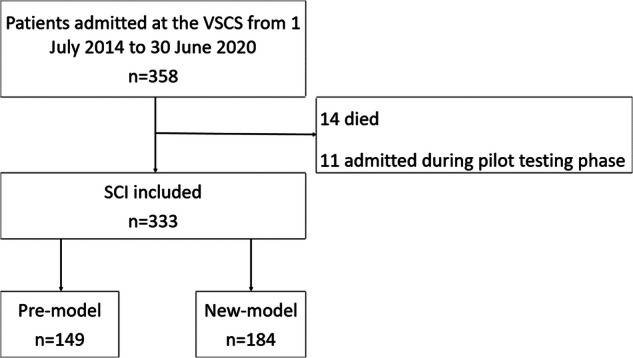
Flow chart with reasons for exclusion.

Three hundred and fifty-eight eligible patients with SCI were registered on the VSTR from 1 July 2014 to 30 June 2020. Fourteen people died during their acute hospital stay, and 11 people were admitted during the pilot testing phase for the new model of care. These 25 people were excluded, leaving 333 cases for analysis; 149 cases in the pre-model of care group and 184 cases in the post-model of care group (Fig. 1).

Individuals were predominantly male (77%), and the mean (SD) age was 48.3 (20.1) years. Demographic and baseline characteristics between the model of care groups are shown in Table 1. There was no association between demographic and SCI characteristics and care group except for the lower proportion of individuals receiving their definitive acute hospital care at the VSCS, and a higher ISS in the new model of care group (Table 1).

**Table 1:** Demographic and clinical characteristics of the cohort.

Characteristic	Pre model of care	New model of care	*p*-value
	(*n* = 149)	(*n* = 184)	
Age, mean (SD) years	48.0 (20.1)	48.9 (19.7)	0.68
Sex			0.69
Male	113 (75.8%)	143 (77.7%)	
Female	36 (24.2%)	41 (22.3%)	
Charlson Comorbidity Index weight			0.48
0	86 (57.7%)	109 (59.2%)	
1	35 (23.5%)	49 (26.6%)	
>1	28 (18.8%)	26 (14.1%)	
Quintile of socioeconomic status			0.14
1 (most disadvantaged)	21 (14.5%)	41 (22.9%)	
2	26 (17.9%)	33 (18.4%)	
3	35 (24.1%)	31 (17.3%)	
4	24 (16.6%)	37 (20.7%)	
5 (least disadvantaged)	39 (26.9%)	37 (20.7%)	
Remoteness of residence			0.99
Major cities	90 (62.1%)	110 (61.8%)	
Inner regional	41 (28.3%)	51 (28.7%)	
Outer regional and remote	14 (9.7%)	17 (9.6%)	
Cause of injury			0.83
Transport	57 (38.3%)	71 (38.6%)	
Low fall (≤1 m or from standing height)	31 (20.8%)	44 (23.9%)	
High fall (>1 m)	37 (24.8%)	45 (24.5%)	
Other cause	24 (16.1%)	24 (13.0%)	
Intent of injury			0.15
Unintentional	145 (97.3%)	173 (94.0%)	
Intentional or intent could not be determined	4 (2.7%)	11 (6.0%)	
Compensable case			0.06
No	110 (75.3%)	120 (65.9%)	
Yes	36 (24.7%)	62 (34.1%)	
Nature of SCI			0.51
Cervical - incomplete/contusion	76 (51.0%)	83 (45.1%)	
Cervical - complete	26 (17.4%)	41 (22.3%)	
Thoracic - incomplete/contusion	17 (11.4%)	17 (9.2%)	
Thoracic or lumbar - complete	30 (20.1%)	43 (23.4%)	
ISS, median (IQR)	20 (16–29)	25 (17–33)	0.002
Inter-hospital transfer			0.29
No	16 (10.7%)	27 (14.7%)	
Yes	133 (89.3%)	157 (85.3%)	
Acute care definitive hospital			0.02
Major trauma Service	6 (4.0%)	20 (10.9%)	
Victorian Spinal Cord Service	143 (96.0%)	164 (89.1%)	
Intensive care unit stay			0.08
No	81 (54.7%)	118 (64.1%)	
Yes	67 (45.3%)	66 (35.9%)	
Length of stay, median (IQR) days	18.7 (10.8–29.1)	17.7 (9.3–33.9)	0.90
Discharge destination			0.35
Inpatient rehabilitation	133 (89.3%)	158 (85.9%)	
Other	16 (10.7%)	26 (14.1%)	

The proportion of individuals who experienced a subsequent ED presentation for UTI was lower in the new model of care phase while the prevalence of readmission and overall hospitalisation in the first 2 years after injury was not different (Table 2).

**Table 2:** Subsequent ED presentations and hospital admissions for urinary tract infection.

Outcome	Pre model of care	New model of care	*p*-value
	(*n* = 149)	(*n* = 184)	
Subsequent ED presentation for UTI			0.04
No	139 (93.3%)	180 (97.8%)	
Yes	10 (6.7%)	4 (2.2%)	
Median (range) presentations if yes	1 (1–5)	1 (1-2)	
Subsequent admission for UTI			0.44
No	135 (90.6%)	171 (92.9%)	
Yes	14 (9.4%)	13 (7.1%)	
Median (range) admissions if yes	1 (1–10)	1 (1–2)	
Subsequent hospitalisation for UTI			0.12
No	130 (87.3%)	170 (92.4%)	
Yes	19 (12.7%)	14 (7.6%)	
Median (range) hospitalisations if yes	1 (1–15)	1 (1–3)	

**Fig. 2:**
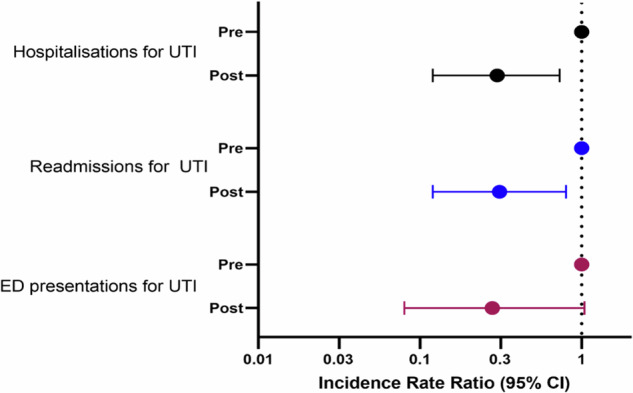
Association between study phase and number of subsequent hospitalisations for UTI.

The rate of ED presentation was not significantly lower following the introduction of the new model of care (IRR 0.21, 95% CI: 0.08, 1.04, Fig. 2). The rate of readmission (IRR 0.21, 95% CI 0.12, 0.80), or any hospitalisation (IRR 0.3, 95% CI 0.12, 0.73), for UTI was lower following the introduction of the new bladder management model of care (Fig. 2). This reflects the lower number of repeat readmissions in the post phase (Table 2).

## Discussion

This was the first study to compare the rate of ED presentations, admissions and hospitalisations after the transition from IDC as primary bladder management to ISC. In this study, the transition to ISC as the primary mode of bladder management was associated with lower rates of readmission and hospitalisation for UTI.

Re-hospitalisation rates for SCI have been demonstrated to be between 36–45% within the first-year post injury and approximately 30% for people with a chronic SCI (>1year) [4]. Of these 32% of presentations are associated with UTIs [4]. Our results are consistent with current literature recommending the use of ISC as gold standard bladder management [11, 16–18] to reduce the risk of UTIs and subsequent need for hospitalisation.

Despite the significant reduction in hospitalisations for UTI following implementation of a new model of bladder management, UTIs still occurred. Urinary tract infections remain one of the most common and distressing conditions for individuals living with a neurogenic bladder and have significant consequences for QoL and health outcomes [8, 14, 17, 24, 25]. Rates as low as 1–3 patient reported UTIs per year can significantly worsen QoL. Compared to individuals who have had no UTIs a patient’s odds of experiencing a worse quality of life increases as the rate of patient reported UTIs increases [25]. UTIs are the most common cause rehospitalisations in SCI [6]. Gabbe and Nunn (2016) found that UTI accounts for 61% of all ED costs for SCI. By reducing the number of ED presentations and subsequent hospitalisation it reduces the financial and resource burden on the healthcare system. Krause et al. [26] also found that the number of hospital bed days over the previous year was a significant predictor of increased mortality rates. Future work should focus how to reduce the number of UTIs for ISC and address potential factors impacting this rate.

A strength of this study was determining the effect implementing a new model of care had on the rate of ED presentations, admissions and hospitalisation. Previous studies have focused on the rate of UTIs in people using certain bladder management strategies [2, 17], or the rate of person reported UTIs [25], but observational studies of the impact of changing models of care are few. By understanding how a change in care impacts the hospital it helps to inform direction of care and resources. Nevertheless, there were limitations to this study. The study was observational before and after study and therefore only association and not causation could be inferred. Additionally this was a single site observational study, patients could have been admitted to hospitals other than the Austin for management of a UTI which would not have been captured in the data. Diagnosis of a UTI was made based on a patient’s presentation or admission being coded using the appropriate ICD-10-AM code. Previous data from the United States suggests this approach results in a high positive predictive value for diagnostic accuracy [27], although supporting data from Australia is not available. While ICD-10-AM coding is performed by trained coders in Australia, and there are robust audit processes for coding, our approach may have lead to over- or under-representation of the incidence of a UTI. While a randomised controlled trial (RCT) would have enabled assessment of causation, an RCT requires clinical equipoise as a foundation for justification. As there was already substantial evidence that ISC was the gold standard for clinical practice [11, 16–18], the justification for a clinical trial was low. ISC would not be indicated in all SCI people, though the case-mix between the phases was comparable, and a change in case-mix was unlikely to explain the findings.

The introduction of the new model of care for bladder management at the VSCS may have been associated with lower rates of ED presentation and hospitalization for UTI. A prospective study is required confirm these findings.

## Data Availability

Requests for access to data from the Victorian State Trauma Registry require approval from the data custodians, who can be contacted at susan.mclellan@monash.edu or at the following URL: http://www.med.monash.edu.au/assets/docs/sphpm/2016_nov_vstr_data_access_guidelines.pdf.
